# Retinal Progenitor Cells Exhibit Cadherin-Dependent Chemotaxis across Transplantable Extracellular Matrix of In Vitro Developmental and Adult Models

**DOI:** 10.1155/2023/1381620

**Published:** 2023-08-24

**Authors:** Miles W. Markey, Caroline D. Pena, Tadmiri Venkatesh, Li Cai, Maribel Vazquez

**Affiliations:** ^1^Department of Biomedical Engineering Rutgers, The State University of New Jersey, 599 Taylor Road, Piscataway, NJ 08854, USA; ^2^Department of Biology, The City College of New York, 140^th^ and Convent Ave, New York, NY 10032, USA

## Abstract

Retinal degeneration is an escalating public health challenge, as diseases such as age-related macular degeneration, diabetic retinopathy, and retinitis pigmentosa cause irreversible vision loss in millions of adults each year. Regenerative medicine has pioneered the development of stem cell replacement therapies, which treat degeneration by replacing damaged retinal neurons with transplanted stem-like cells (SCs). While the collective migration of SCs plays critical roles during retinal development, our understanding of collective SC behaviors within biomaterials transplanted into adult tissue remains understudied. This project examines the potential therapeutic impacts of collective SC migration during transplantation by correlating the expression of cadherin, cell-cell cohesion molecules that maintain intercellular communication during development, with receptor proteins of chemoattractant molecules prevalent in degenerated adult tissue. Experiments examine these well-conserved biomechanisms by using two different model organisms: *Drosophila melanogaster*, a seminal model for retinal development, and *Mus*, an important preclinical model for transplantation. Results indicate that SCs from both animal models significantly upregulate cadherin expression to achieve more directed collective migration towards species-specific chemoattractants and exhibit longer distance motility upon different extracellular matrix substrates.

## 1. Introduction

Retinal diseases are public health challenges that impact growing populations each year [1]. Degenerative disorders, such as age-related macular degeneration, diabetic retinopathy, and retinitis pigmentosa, cause progressive vision loss in millions of aging adults, worldwide (reviewed in [2]). Regenerative medicine offers promising strategies to treat many retinal disorders through cell replacement therapies, where donor stem-like cells (SCs) are transplanted via biomatrixes into adult host to replace groups of damaged or degenerated neurons [3, 4].

**Figure 1:**
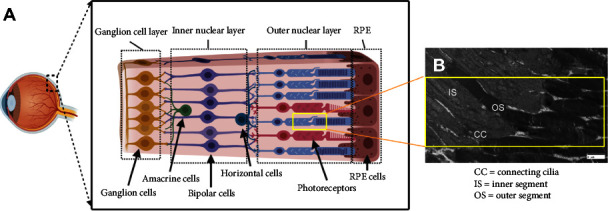
Schematic of an adult mammalian retina. (A) The mature retina is composed of multiple cell layers (from right to left): retinal pigment epithelium (RPE), outer nuclear layer (containing rod and cone photoreceptors), inner nuclear layer (containing amacrine, bipolar, and horizontal cells), and the ganglion cell layer. (B) Image of outer nuclear layer gathered via transmitted electron microscopy (TEM) indicating where stem-like cells (SCs) are used as photoreceptor cell replacements. Image illustrates the photoreceptor inner segment, outer segment, and connecting cilia as marked (scale = 2 *μ*m).

Vision depends on retinal neurons that enable the phototransduction of light to produce images of objects in the brain. Incident light is absorbed and transduced into electrical signals by rod and cone photoreceptor neurons. As shown in Figure 1, photoreceptors are tightly packed within the outer nuclear layer of a healthy retina but will exhibit larger, nonuniform spacing in degenerated tissue. Rods and cones synapse with interneurons of the inner nuclear layer (e.g., bipolar, horizontal, and amacrine cells), which in turn network with cells of the ganglion layer. Ganglion neurons then transmit these signals along the optic nerve to the visual cortex. Cell damage in any portion of this sophisticated network leads to vision loss, as mature retinal neurons cannot self-repair and their degeneration or injury produces irreversible damage [5, 6].

The functional integration of donor cells needed to re-establish vision is decidedly complex in large part because the behaviors and responses of matrix-transplanted SCs to signals from adult tissue environments remain incompletely understood. Moreover, the integration of SCs relies upon multiple critical and often concurrent cell processes, in situ. Transplanted SCs must first survive surgical insertion and migrate within the retinal host to position themselves into damaged regions of mature cellular networks [7]. In tandem, donor SCs must adapt to rapid changes in cell fate along highly specialized, retinal differentiation pathways [8]. Through these intricate biological processes, transplanted SCs must ultimately establish synaptic communication with functional native neurons to transmit the photonic signals needed for vision.

Contemporary tissue engineered biomaterials have dramatically advanced survival of donor SCs in the visual system by mimicking native and degenerated matrix [9]. Similarly, differentiation pathways of donor SCs have been enriched by materials that harvest specialized, transplantable cells from new autologous and xenograft sources [10] and by targeting families of molecules specific to retinal phenotypes [11]. By contrast, the migration of SCs within and upon transplantable biomaterials and into the retinal host remains surprisingly underexplored [12]. The factors that contribute to extremely low numbers of motile cells, as well as their low-distance and nondirectional migration, remain understudied [7, 13, 14].

While the movement of retinal SCs along gradients of bound and diffusible factors has been well-examined during development using genetic models of invertebrate species to mammals [15, 16], the physiochemical environment of the mature retina into which donor SCs are transplanted is dramatically different from that of the developing retina. Adult tissues produce entirely different combinations of cytokines and factors, as well as extracellular matrix (ECM) with wide-ranging rheological properties [17]. The impact of collective SC migration within transplantable materials, and into host tissue itself, remains significantly unexamined in transplantation, despite the ubiquitous and essential collective behaviors exhibited by SCs in endogenous environments of developing tissue (reviewed in [18]).

Collective chemotaxis relies upon both asymmetric binding of ligands to spatially detect concentration gradients and cell-cell cohesion to couple cytoskeletal movements of adjacent cells [19, 20]. Cell adhesion molecules from the cadherin family have been shown to influence collective responses to changes in ECM stiffness and mechanical forces, as well as to morphogenic cues that trigger differentiation and cell fate [21]. Transplantation projects using biomaterials have not examined relationships between cell cohesion mechanisms and collective chemotactic pathways to understand and/or predict SC responses to cues from the mature retina. Moreover, the fundamental knowledge gained of these well-conserved mechanisms using developmental models, such as fruit flies, frogs, and fish [22, 23], has not been applied to advance transplantation in vertebrate animals.

Our group has previously reported that retinal SCs exhibit collective chemotaxis, with preferential size dependent on the nature and strength of the chemical gradients imposed [24, 25]. We have further demonstrated that motile donor SCs respond to different chemotactic factors and pathways in adult versus immature retina [26, 27]. The current study examined the interplay between chemotactic receptor proteins and cadherin molecules within retinal SCs from different species to examine how fundamentally conserved behaviors can be explored to advance stem cell therapy. Experiments used retinal progenitors derived from *Drosophila melanogaster*, a seminal retinogenesis model, and SCs of *Mus*, a common rodent model of retinal transplantation. Results illustrate that cells from both animals migrated most directionally, and in larger numbers, when cadherin molecules were upregulated in concert with chemotactic receptors across a variety of ECM. These findings highlight conserved mechanisms of cell-cell cohesion and collective migration that may be exploited to develop migration-targeted biomaterials and therapies in the retina.

## 2. Methods

### 2.1. Cell Culture

#### 2.1.1. *Drosophila melanogaster*

The D17 cell line (ML-DmD17-c3 (DGRC Stock 107; https://dgrc.bio.indiana.edu//stock/107; RRID: CVCL_Z772)) was used to model *Drosophila* progenitors (DPs) from the imaginal disc in this study, as done previously by our group [24, 25] using established protocols [28]. First, cell culture media was prepared under sterile conditions using 88.75% v/v Schneider's insect medium (ThermoFisher Catalog #21720024) and 10% v/v fetal bovine serum heat inactivated at 55°C (ThermoFisher Catalog #10099141). Note that most commercially available, heat-inactivated FBS is treated at temperatures ≥65°C, which is unsuitable for long-term D17 cell culture. These solutions can be heat-inactivated using a 55°C water bath for 1 hr, 1% v/v antibiotic/antimycotic (ThermoFisher Catalog #15240096), and 0.25% v/v human insulin (ThermoFisher Catalog #12585014). Next, flasks of D17 cell cultures obtained from the DGRC were allowed to settle for 6–12 hrs, while all but 5 mL of media was withdrawn and reserved for future use. Once cells had reached confluency in 1-2 days, the medium was aspirated and the flask was washed with 3 mL of enzyme-free Hank's balanced salt solutions cell dissociation buffer (ThermoFisher Catalog #13151014). This cell dissociation buffer was then aspirated, and 3 mL of fresh cell dissociation buffer was added while cells were incubated at 25°C for 25 minutes. The cell dissociation buffer was later aspirated, and 3 mL of fresh D17 growth medium was added. The medium was vigorously pipetted to dislodge cells from the flask, and cells were passaged into a fresh T-25 flask (VWR Catalog #29185-078) at a ratio of about 1 : 2 (i.e., the confluency of the new flask is approximately 50%). Flasks were then incubated at 25°C and passaged again after confluency 3-4 days later.

#### 2.1.2. *Mus*

The commercial R28 cell line (Kerafast, MA, EUR201) was used to model rodent retinal progenitors (MPs) for this study, as done previously by our group and others [29, 30]. R28 growth medium was prepared under sterile conditions using 89% Dulbecco's Modified Eagle's Medium (DMEM; ATCC Catalog #30-2002), 10% Fetal Bovine Serum, and 1% Pen-Strep (FBS; ThermoFisher Catalog #26140087). Cells were grown in ventilated T-25 flasks (VWR Catalog #29185-300) until confluency, typically 4-5 days. Growth medium was then aspirated, and cells were incubated with 3 mL of Accutase (ThermoFisher Catalog #A1110501) at 37°C for 5 minutes to dislodge adherent cells. The Accutase was then removed, placed in a 15 mL centrifuge tube, and centrifuged at 1500 RPM for 5 minutes. The supernatant was aspirated, and the cell pellet was resuspended in 3 mL of fresh R28 growth medium. The cells were then reseeded in a new T-25 flask at 10^6^ cells per mL and incubated at 37°C and 5% CO_2_ until confluency.

### 2.2. Reagents

#### 2.2.1. Chemoattractants

Cells were exposed to a panel of growth factors and different extracellular matrix (ECM) compounds listed in Table 1 and chosen based on previous work from our lab and others [15, 25]. The growth factors studied were stromal-derived growth factor 1-alpha (SDF-1*α*), epidermal growth factor (EGF), insulin-like growth factor 1 (IGF-1), fibroblast growth factor-8 (FGF-8), pyramus (Pyr), brain-derived neurotrophic factor (BDNF), and insulin (IN). ECMs used were laminin (LM), poly-L-lysine (PLL), and matrigel (MAT).

#### 2.2.2. Antibodies

Proteins on both DPs and MPs were examined using commercial antibodies for staining. For the study of DPs, a rat anti-Ncad primary antibody (Developmental Studies Hybridoma Bank DN-Ex #8) and rat anti-Ecad primary antibody (Developmental Studies Hybridoma Bank DCAD2) were used. The secondary antibodies were goat antirat IgG + AlexaFluor 488 conjugate (ThermoFisher Catalog #A48262). FGFR (breathless, Btl) of DPs was measured using rabbit antibreathless (Abcam Catalog #155960) as the primary antibody and goat antirabbit IgG + AlexaFluor 488 (ThermoFisher Catalog #A32731) as the secondary antibody.

The study of MPs used a mouse anti-Ncad primary antibody (ThermoFisher Catalog #33-3900) and mouse anti-Ecad primary antibody (ThermoFisher Catalog #MA5-15717). The secondary antibodies were goat antimouse IgG + AlexaFluor 488 conjugate (ThermoFisher Catalog #A-11001).

### 2.3. Migration Assays

Transwell Boyden chamber assays (Sigma-Aldrich Catalog #ECM506) were used to examine the motility of each cell type toward different growth factor concentrations. Cells were seeded in the upper chamber of the well assay and allowed to settle on the upper surface of the membrane overnight. Growth factor solutions were then added to the media in the lower reservoir, and cells were left to migrate for 2 hours through the transwell membrane, as described previously by many groups [31].

A parameter called the cell migration index, CMI, was defined to numerically compare the results of motile cells in response to different chemoattractants and control experiments (medium only). CMI is defined as the ratio of the average numbers of motile cells in response to an exogenous growth factor and the average number of motile cells in control wells, as shown in the following equation:

(1) $$\begin{array}{c} {CMI}_{i}=\frac{n_{i}}{N_{control}}\text{,} \end{array}$$

where the subscript *i* denotes the reagent in a specific well measured, *n*_*i*_ represents the number of motile cells in that well, and “*N*_control_” is the average number of motile cells in control wells, per experiment.

### 2.4. Cell Staining and Immunocytochemistry (ICC)

#### 2.4.1. Cells

Motility and viability of DPs and MPs were determined using LIVE/DEAD assays with Calcein-AM (5 *μ*M, ThermoFisher Catalog #C1430) and transwell assays. The contents of the lower reservoir of transwell assays were aspirated and replaced with an equal volume of media containing Calcein-AM. Cells were exposed to Calcein-AM for 1 hr following established cell viability protocols [32], and the lower transwell surface was imaged to count the number of live cells that had migrated in each well.

#### 2.4.2. Proteins

Immunocytochemistry (ICC) staining was performed on both cell types following established protocols [25]. Briefly, cells were passaged at mid-exponential growth and seeded in borosilicate well plates (ThermoFisher Catalog #155361) in standard cell culture media, described earlier for DPs and MPs, and left to adhere for 4–12 hr. The media was then aspirated, and cells were fixed in 4% PFA for 20 minutes at room temperature (25°C), using enough PFA to just coat the bottom of each well (approximately 500-*μ*L). After fixing, the PFA was aspirated and 500 *μ*L of antibody blocking buffer (DPBS + 3% goat serum + 2% BSA + 0.05% Triton-X) was added to each well. The wells were kept at room temperature (25°C) for 2 hrs to allow the blocking buffer to fully permeabilize the cells. While blocking, a solution of 3 *μ*g/mL primary antibodies (anti-Ncad and anti-Ecad) were prepared in Dako antibody dilution buffer (Agilent Product #S3022). After 2 hrs, the blocking buffer was aspirated, and 500 *μ*L of the primary antibody solution was added to each well. The cells were left at room temperature overnight. The following day, a secondary antibody solution was prepared under low-light conditions to prevent photobleaching of the secondary antibody. The primary antibody solution was then aspirated from the wells, and the wells were washed 3X with DPBS for 5 minutes, each. The wells were aspirated, and 500 *μ*L of the secondary antibody was added and left to sit at room temperature for 1.5 hrs. The secondary antibody solution was then aspirated, and the wells are washed 3X with DPBS for 5 minutes, each. A 500 *μ*L of a 75 nM solution of DAPI (ThermoFisher Catalog #D1306) was added to the wells for 5 minutes, and the wells were washed with DPBS for 5 minutes. Experiments used comparable cell densities of ∼10^4^ cells/mL in each test (*n* = 4).

### 2.5. Experimental Setup

Molecular expressions of N-cadherin and E-cadherin were measured via ICC experiments. DPs were seeded in well plates and exposed to 10 ng/mL of either mammalian (Gibco #PMG0034) or invertebrate (Gibco Cat #PHG0184) FGF for 2 hrs. DPs were subsequently stained for *Drosophila* FGFR (breathless, Btl) and analyzed to assess Btl expression. DPs were seeded in well plates and exposed to appropriate concentrations of *Drosophila* growth factors for 2 hrs. DPs were then fixed and stained separately for N-cadherin and E-cadherin. MPs were seeded in well plates and exposed to appropriate concentrations of rodent growth factors for 2 hrs. The cells were then fixed and stained separately for both N-cadherin and E-cadherin using the MP antibodies described earlier. MPs were seeded in wells that had been coated with laminin, poly-L-lysine, matrigel, and control (no ECM coating) following product-specific protocols. The cells were then fixed and stained for E-cadherin and N-cadherin following the previously established ICC protocols without the addition of growth factor. All results were analyzed using analyses protocols described below.

### 2.6. Imaging and Data Analysis

Fluorescent imaging was performed using the Leica THUNDER Imager at 40x magnification with an oil immersion objective. The wells were then imaged concurrently using both a Texas Red (TXR) and DAPI filter cube to obtain images showing both N-cadherin expression and DAPI. ImageJ software was then used to measure the average fluorescent intensity of the TXR channel of each image. A custom imaging processing algorithm to quantify normalized cadherin expression via fluorescent intensity was developed using MATLAB. The algorithm first separates the channels of the stained images (RGB format), where the red channel contains solely the cadherin expression information and the blue channel contains solely the DAPI staining information. A cell-counting algorithm (adapted from [33]) was used to count the number of cell nuclei in the DAPI channel of each image, while the average intensity of the red channel was used to determine the average cadherin expression of each image, as per the following equation:

(2) $$\begin{array}{c} normalized\;cadh\;erin\;expression\;level=\frac{R_{average}}{N_{nuclei}}\text{,} \end{array}$$

where “*R*_average_” is the average fluorescent intensity of the activated channel and “*N*_nuclei_” is the number of nuclei counted from the DAPI channel in each image. This value was then normalized to the number of cell nuclei in the image, giving a final value of normalized cadherin expression per cell for each stained image.

### 2.7. Transmission Electron Microscopy (TEM)

Isolated fresh mouse retina tissue samples were rinsed in cacodylate buffer (Electron Microscopy Sciences, CAS #11650) and fixed in buffered 1% osmium tetroxide (Electron Microscopy Sciences #MFCD00011150). Samples were then dehydrated in a graded series of acetone (Sigma-Aldrich #34850) and embedded in Epon resin (Electron Microscopy Sciences #EMbed 812). Afterwards, 1 *μ*m thick sections were cut, affixed upon glass slides, and stained with 1% methylene blue (Sigma-Aldrich #122965-43-9). From these samples, 90 nm thick sections were placed upon sectioned copper grids (Electron Microscopy Sciences, CAS #102100-060) and stained with saturated solutions of uranyl acetate and lead citrate (Electron Microscopy Sciences, CAS #512-26-5). Images were captured using an AMT XR111 digital camera (AMT XR111) on a Philips CM12 transmission electron microscope (Philips CM12).

### 2.8. Statistical Analyses

One-way ANOVA was used to analyze statistical significance among parametric datasets. Each dataset was gathered from a minimum of four biological replicates using *n* = 4 to *n* = 12 wells, per condition, with 10–15 images collected per well. Values are reported using statistical mean and standard deviation, while the post hoc Tukey test was used to determine significance between conditions. Significance via *p* values of <0.05 were denoted by an asterisk, ^*∗*^, while values of *p* < 0.01 were marked with a double asterisk, ^*∗∗*^.

## 3. Results

Experiments of this project examined how cell-to-cell and cell-to-matrix interactions are conserved across retinal progenitors derived from *Drosophila melanogas*ter and *Mus* animal models. The study examined collective SC behaviors using a panel of ECM substrates and different groups of chemoattractant molecules to identify how cohesive mechanisms conserved across species may be explored to develop new transplantable biomaterials.

### 3.1. Cell Aggregation and Cadherin Activation Observed in All Retinal Progenitors

**Figure 2:**
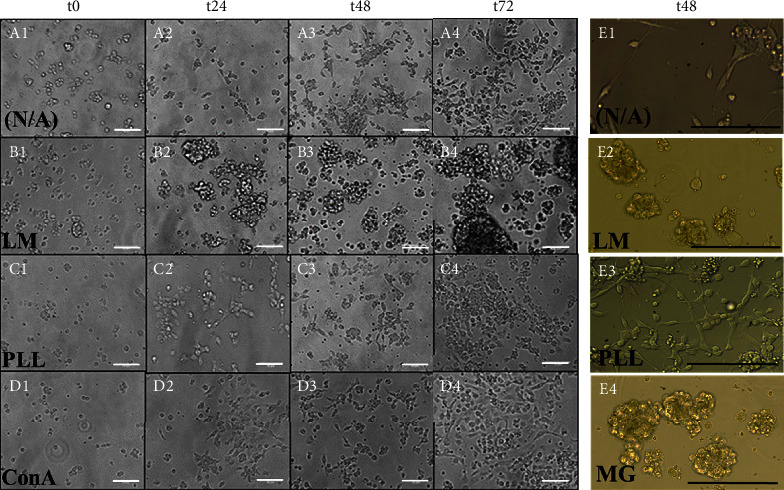
Stem-like cells exhibit patterns of adhesion upon different extracellular matrices. *Drosophila* progenitor cells (DPs) observed upon (A) uncoated Petri dishes (N/A; *n* = 4), (B) laminin (LM4; *n* = 10), (C) poly-L-lysine (PLL; *n* = 6), and (D) concanavalin (Con-A; *n* = 12) coated Petri dishes at time points t0 (2 hours after seeding to allow for adhesion), t24 (24 hours), t48 (48 hours), and t72 (72 hours) (scale bars: 50 *μ*m). (E) Mouse progenitor cells (MPs) adhered upon uncoated Petri dishes (N/A; *n* = 8), laminin (LM; *n* = 8), poly-L-lysine (PLL; *n* = 8), and matrigel (MG; *n* = 8) coated Petri dishes at time point t48 (scale bars: 100 *μ*m).

The first series of experiments examined the cell aggregation and collective adhesion of progenitors from *Drosophila melanogaster* (DPs) and *Mus* (MPs) upon different ECM substrates common to each species. Figures 2 (A)–(D) illustrate the clustering behavior of DPs after seeding upon laminin (LM; *n* = 10), poly-L-lysine (PL; *n* = 6), and concanavalin A (ConA; *n* = 12) substrates over 72 hours. Spontaneous cluster formation was observed in all cases and correlated with the survivability data collected previously by our group [24, 34].  Figure 2 (E) illustrates similar clustering behavior of MPs over 72 hours after seeding upon laminin (LM; *n* = 8), poly-L-lysine (PLL; *n* = 8), and matrigel (MG; *n* = 8) substrates.

**Figure 3:**
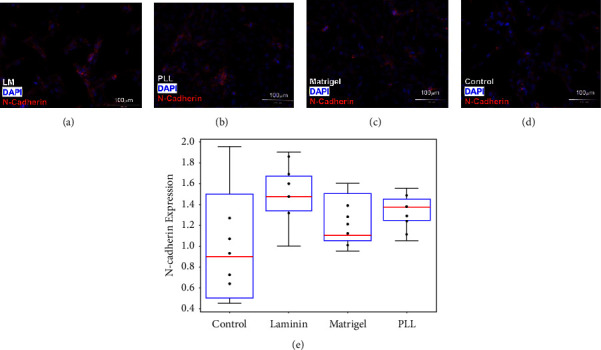
N-cadherin expression in cultured *Mus* retinal progenitors seeded on (a) laminin (LM), (b) poly-L-lysine (PLL), (c) matrigel (MG), and (d) control (no ECM coating). Red indicates N-cadherin and blue indicates DAPI nuclear stain. (e) N-cadherin expression levels for each group normalized to control show significant changes in N-cadherin expression for LM (*p* < 0.05) but not on MG or PLL. A sample of *n* = 5 wells was used per condition.

The next experiments measured the expression of E- and N-cadherin per DP and MP groups.  Figure 3 illustrates differences in the N-cadherin expression of MPs upon laminin (LM; *n* = 6), poly-L-lysine (PLL; *n* = 6), and matrigel (MG; *n* = 6) substrates relative to control. As seen, MPs illustrated the largest expression of N-cadherin when planted upon LM (*p* < 0.05), but not statistically different upon MG or PLL (*p* > 0.05). No significant changes in E-cadherin expression (*p* > 0.05) were observed in MPs seeded on each ECM substrate relative to control (no coating) (data not shown). By contrast, DPs did not exhibit N-cadherin expression (data not shown) but did express E-cadherin upon the ConA substrate, as previously shown by our group [34].

### 3.2. Chemotactic Migration Is Growth Factor and Species-Specific

Tests examined whether DPs and MPs responded via motility to species-specific homologs of the same growth factor. Activation of the *Drosophila* FGF receptor, breathless (Btl), was measured in DPs after exposure to equal concentrations of mammalian FGF-8 and its *Drosophila* homolog, pyramus (Pyr). The data illustrate that the average fluorescence intensity of activated Btl per cell was 1.6-fold higher (*p* < 0.05) than control (media only) when exposed to Pyr but downregulated 0.6-fold when exposed to mammalian FGF-8. No differences were measured in responses to other homologs of either cell type (Supplemental Figure 1).

**Figure 4:**
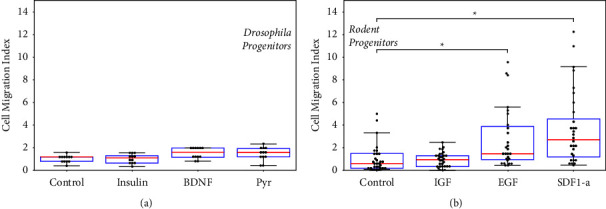
Measurement of the chemotaxis of stem-like cells. (a) Numbers of motile *Drosophila* progenitors (DPs) exposed to concentration gradients of insulin (*N* = 12), brain-derived neurotrophic factor (BDNF, *N* = 12), and pyramus (Pyr, *N* = 12) normalized to control (media only, *N* = 12). (b) Numbers of motile *Mus* progenitors (MPs) in response to concentration gradients of insulin-like growth factor 1 (IGF-1, *N* = 30), epidermal growth factor (EGF, *N* = 27), and stromal cell-derived factor 1-alpha (SDF-1*α*, *N* = 28) and normalized to control (media only, *N* = 30). Bars indicate statistically significant increases compared to control (^*∗*^*p* < 0.05).

The motility of both DPs and MPs was next measured in response to a panel of species-specific growth factors using transwell assays and the cell motility index (CMI) of equation (1). DPs were incubated with insulin, brain-derived growth factor (BDNF), and pyramus (Pyr), the *Drosophila* homolog of fibroblast growth factor (FGF-8). As shown in Figure 4(a), DPs exhibited a 1.4-fold increase in numbers of motile cells (*p* < 0.05) toward concentration gradients of both BDNF and Pyr relative to control (media only), but approximately the same level of motility toward Insulin as control (*p* > 0.05). As seen, the overall values were CMI <2 for all testing. MPs were similarly incubated with insulin-like growth factor 1 (IGF-1), epidermal growth factor (EGF), and stromal-derived growth factor alpha (SDF-1*α*) to evaluate chemotaxis.  Figure 4(b) illustrates that MPs exhibited larger CMI values to denote 5-fold increases in numbers of motile cells (*p* < 0.05) in response to signaling from both EGF and SDF1-*α* gradients relative to control (media only). No measurable change in MP motility toward IGF-1 gradients (*p* > 0.05) was observed.

### 3.3. Cadherin Activation Correlates to Progenitor Clustering in Response to Chemoattractants

**Figure 5:**
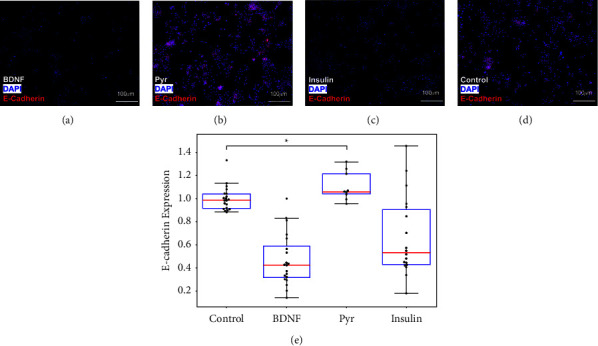
E-cadherin expression in *Drosophila* progenitors (DPs) treated with (a) BDNF (*N* = 20), (b) Pyr (*N* = 10), (c) insulin (*N* = 18), and (d) control (media only, *N* = 20). Red indicates E-cadherin molecules and blue indicates DAPI nuclear staining. (e) E-cadherin expression levels for each group normalized to control. Significantly higher E-cadherin expression is observed in the Pyr-treated group compared to control (*p* < 0.05).

The next set of experiments measured the expression of E-cadherin and N-cadherin in response to the chemotactic factors studied for both DPs and MPs.  Figure 5 illustrates that DP cells exposed to extracellular BDNF and insulin expressed significantly lower levels (*p* < 0.05) of E-cadherin than control (media only). By contrast, DPs exposed to the highest chemotactic factor, Pyr, expressed levels of E-cadherin that were 1.1-fold (*p* < 0.05) than those of control (media only). In addition, tests examined the DP expression of N-cadherin in response to these factors to measure no significant changes in N-cadherin expression compared to control (*p* > 0.05) (Supplemental Figure 2).

**Figure 6:**
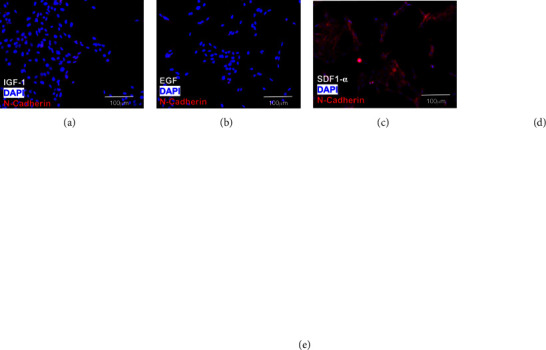
N-cadherin expression in *Mus* retinal progenitors (MPs) treated with (a) IGF-1 (*N* = 23), (b) EGF (*N* = 18), (c) SDF1-*α* (*N* = 19), and (d) control (media only, *N* = 24). Red indicates N-cadherin and blue indicates DAPI nuclear stain. (e) N-cadherin expression levels for each group normalized to control illustrating significantly higher N-cadherin expression in the SDF1-*α* treated group compared to control (*p* < 0.05).

Figure 6 illustrates the differences in N-cadherin expression measured in MP cells exposed to the SDF1-*0*, EGF, and IGF-1 growth factors tested earlier. As seen, average N-cadherin expression per MP cell was 3-fold higher (*p* < 0.05) towards SDF1-*α* than in control (media only), but only 0.7-fold that of control in response to EGF and 0.6-fold that of control in response to IGF-1 (*p* > 0.05). As before, tests to examine MP expression of E-cadherin in response to these factors illustrated no significant changes compared to control (*p* > 0.05) (Supplemental Figure 3).

## 4. Discussion

Cell-to-cell cohesion and cell-matrix adhesion are essential components of stem-like cell (SC) behavior, as groups of cells with similar lineage self-aggregate, or cluster, into collective groups to achieve migration, differentiation, and tissue formation [16, 18]. The current project examined cell-to-cell cohesion mechanisms and cell-matrix interactions as potential strategies to enrich the positioning of donor SCs within damaged cellular regions in response to cues from the adult host during transplantation.

SCs derived from both fly and rodent were used to help bridge fundamental mechanisms from developmental biology across species with applications in SC transplantation. Cell-matrix interactions play key roles in the differentiation of retinal progenitors during development [8] and have been explored using transplantable ECM substrates that increase cell survival as well as expression levels of key proteins [35]. Previous studies from our lab have illustrated significant increases in the survival of *Drosophila* progenitors (DPs) and *Mus* progenitors (MPs) when seeded upon select biomaterials [12, 24]. The current project now observed that these same ECMs promote collective behaviors of adherent cells in both DPs and MPs (Figure 2). We further examined cell-cell cohesion across DPs and MPs to show increased cadherin expression in motile cells upon these ECM (Figures 5 and 6). While the migration and differentiation of donor SCs are essential to transplantation, the innate ability and/or tendency of SCs to respond collectively to external signals from adult host remains underexplored [24]. Our comprehensive data suggest that SC-ECM interactions can be used to not only increase the survival of transplanted cells but also to promote collective SC responses that aid functional integration of cell replacements [36]. Experiments further correlated activation of chemotactic receptors with upregulated cadherin expression in SC collective migration upon transplantable ECM. Testing of self-aggregation upon different substrates helped identify the prominent role of cadherin molecules in the collective chemotactic response to species-specific growth factors (Figure 4). Moreover, the data reflected the same biomechanisms of intercellular adhesion across the model species used.

Recent biological models have suggested that collective migration can be driven through the formation of intercellular junctions, as interactions between clustered cells may be needed for the expression of receptor proteins that stimulate migratory signaling pathways [19, 37]. Migration-focused projects [38] have further demonstrated that cell clustering may provide an advantage for collective sensing of exogenous gradients due to the greater change in absolute concentration across a cell cluster than an individual cell. Our current project demonstrated that collective migration and increased expression of intercellular junctions were evident in both DPs and MPs for E- and N-cadherin, the cell adhesion molecule active during retinogenesis per species, respectively. Tests revealed that E-cadherin was upregulated in DPs after exposure to pyramus (the FGF receptor for that model organism), but not after exposure to either BDNF or insulin, despite measured DP chemotaxis in response to those gradient fields (Figure 5). These results are in agreement with independent projects that have reported that pyramus-mediated migration of DPs is E-cadherin dependent [39]. Additional results observed that MPs express significantly higher levels of N-cadherin when exposed to SDF1-*α*, but not when exposed to EGF or IGF-1 (Figure 6). Similar to the E-cadherin results of DPs, these results indicate that SDF1-*α* induced migration of MPs is correlated with N-cadherin [40]. As expected, MPs showed no significant change in E-cadherin expression after exposure to the growth factors tested, while DPs illustrated no significant difference in N-cadherin (Supplemental Figures 2 and 3). These results indicate that cadherin proteins play a prominent role in the collective chemotaxis of progenitors that is conserved across animal models. Activation of cadherin may then be explored in new transplantable materials to promote collective behaviors befitting SC tendencies.

Biomaterials play essential roles in developing migration-targeted strategies able to enrich SC replacement. Such technology will facilitate the biomedical study of concentration-dependent expression of cadherin in response to customized gradients of key biocompounds in the damaged retina [31]. Moreover, the study of dynamic cadherin expression in retinal progenitors would enable correlation between cytoskeletal changes and cell-cell adhesion, further revealing the importance of intercellular junctions employed during development for applications in transplantation. Lastly, stem cell therapies can leverage these advancements to promote desired SC behaviors to aid directed motility within the degenerated host, cell differentiation, and the synaptogenesis needed to restore vision.

## Figures and Tables

**Table 1:** Summary of growth factors and extracellular matrix (ECM) used to examine the migratory behaviors of retinal progenitors cultured from *Drosophila melanogaster* (DPs) and from rodent (MPs).

Growth factor examined (Catalog no., manufacturer)	Concentration	Species reactivity
Stromal-derived growth factor 1-alpha (SDF1-*α*; ThermoFisher Catalog #RP-8658)	100 ng/mL	MPs
Epidermal growth factor (EGF; ThermoFisher Catalog #PHG0311)	20 ng/mL	MPs
Insulin-like growth factor 1 (IGF1; ThermoFisher Catalog #PHG0071)	50 ng/mL	MPs
Fibroblast growth factor-8 (FGF-8; ThermoFisher Catalog #RP-87808)	10 ng/mL	MPs
Pyramus (Pyr; ThermoFisher Catalog #PHG0184)	10 ng/mL	DPs
Brain-derived neurotrophic factor (BDNF; ThermoFisher Catalog #10908-010)	10 ng/mL	DPs
Insulin (IN; ThermoFisher Catalog #RP-10908)	10 ng/mL	DPs
Concanavalin A (ConA; eBioscience, #00-4978-93)	15 *μ*g/mL	DPs
Laminin (LM; ThermoFisher Catalog #23017015)	15 *μ*g/mL	DPs and MPs
Matrigel (MAT; Fisher scientific Catalog #CB-40234A)	10 *μ*g/mL	DPs and MPs
Poly-L-lysine (PLL; Fisher scientific Catalog #A005C)	20 *μ*g/mL	DPs and MPs

## Data Availability

The experimental data used to support the findings of this study are available from the corresponding author upon request.

## Supplementary Materials

Supplementary Materials Data describing the lack of statistical significance between nonspecies-specific growth factors and receptors are provided. Figure S1 highlights the specificity of FGF-8 homologs between *Drosophila* and *Mus*, while Figures S2 and S3 illustrate specific E-cadherin and N-cadherin dependence, per species.
