# Supplementary material for: Retinal Progenitor Cells Exhibit Cadherin-Dependent Chemotaxis across Transplantable Extracellular Matrix of In Vitro Developmental and Adult Models
Source: J Tissue Eng Regen Med. 2023 Aug 24;2023:1381620. doi: 10.1155/2023/1381620 (PMC11919238; doi:10.1155/2023/1381620)

**SUPPLEMENTAL FIGURES**

Data describing the lack of statistical significance between non-species-specific growth factors and receptors is provided. Figure S1 highlights the specificity of FGF8 homologs between Drosophila and Mus, while Figures S2 and S3 illustrate specific E-cadherin and N-cadherin dependence, per species.

**Supplemental Figure 1**: Expression levels of the receptor breathless (Btl) in cultured Drosophila progenitors (DPs) exposed to mammalian FGF-8 and to the Drosophila homolog Pyramus (Pyr), normalized to control (media only). Statistically significant increases in Btl activation are shown in the Pyr-treated group against control ($p<0.05$).

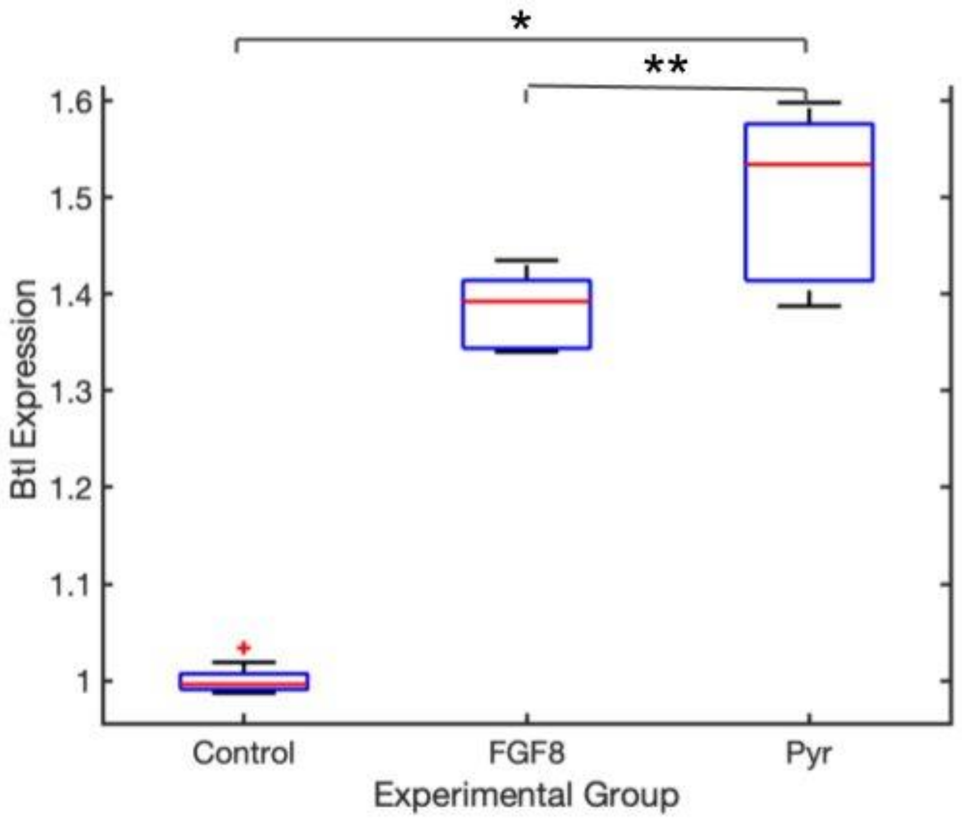

**Supplemental Figure 2**. N-cadherin expression in Drosophila progenitors (DPs) treated with (A) BDNF, (B) Pyr, (C) Insulin, and (D) control (media only). Red indicates N-cadherin molecules and blue indicates DAPI nuclear staining. (E). N-cadherin expression levels for each group normalized to control. No significant changes in N-cadherin expression are observed in any group ($p>0.05$).

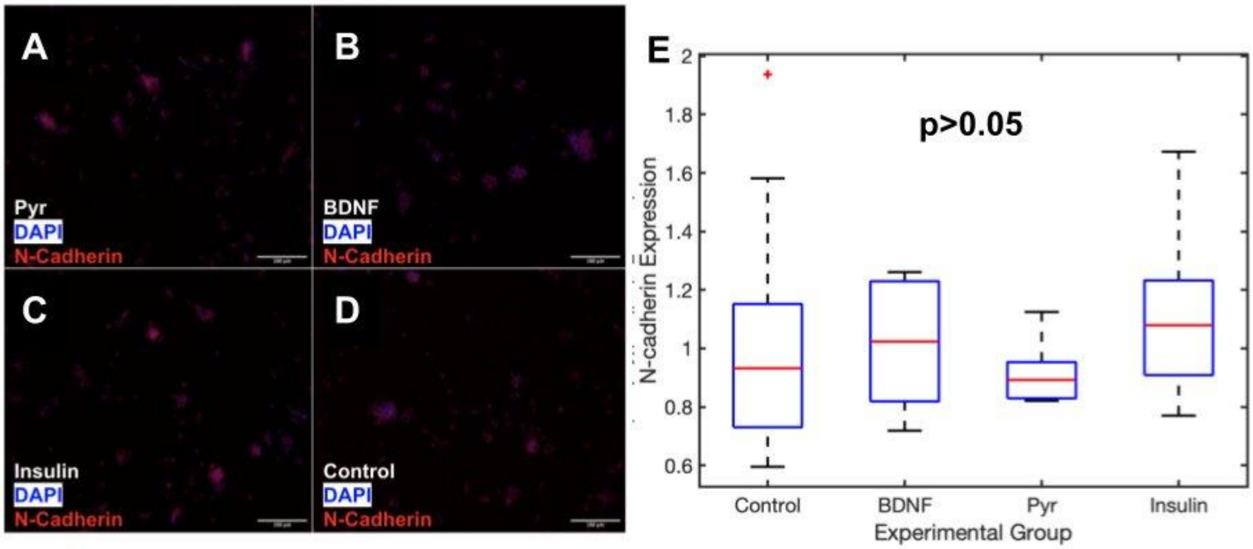

**Supplemental Figure 3**. E-cadherin expression in mus retinal progenitors (MPs) treated with (A) IGF-1, (B) EGF, (C) SDF1-$\boldsymbol{\alpha}$, and (D) control (media only). Red indicates N-cadherin and blue indicates DAPI nuclear stain. (E). E-cadherin expression levels for each group normalized to control illustrating no significant changes in E-cadherin expression are observed in any group (p>0.05).

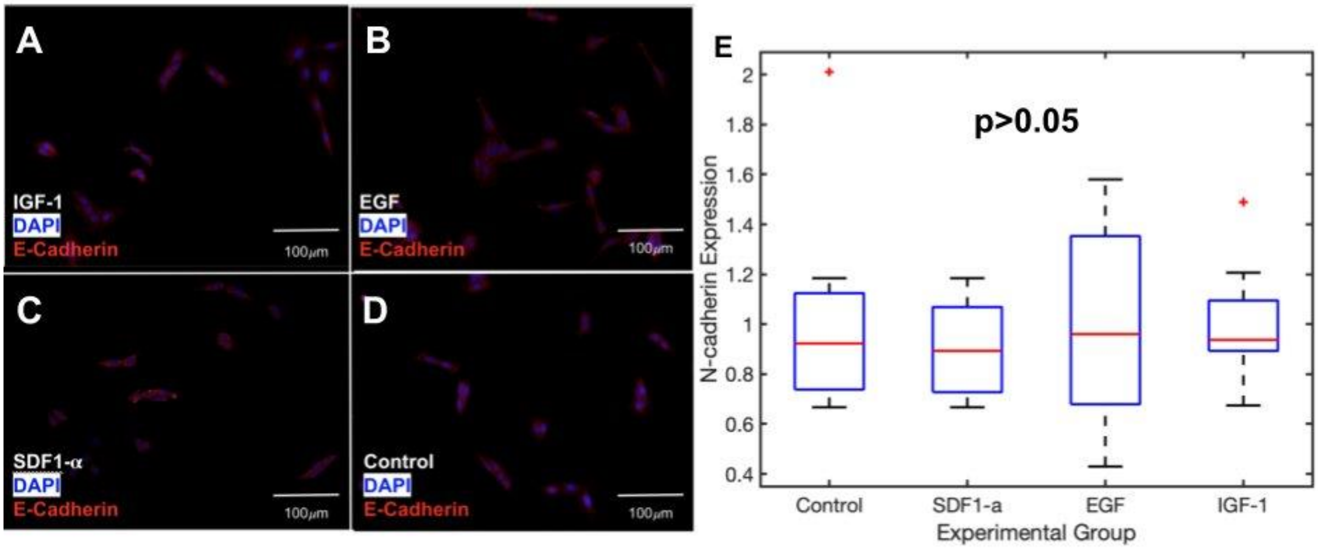

Supplement: Supplementary Materials — Data describing the lack of statistical significance between nonspecies-specific growth factors and receptors are provided. Figure S1 highlights the specificity of FGF-8 homologs between Drosophila and Mus, while Figures S2 and S3 illustrate specific E-cadherin and N-cadherin dependence, per species. [file 1381620.f1.pdf]
